# Counter-Balance Between Gli3 and miR-7 Is Required for Proper Morphogenesis and Size Control of the Mouse Brain

**DOI:** 10.3389/fncel.2018.00259

**Published:** 2018-08-17

**Authors:** Longbin Zhang, Taufif Mubarak, Yase Chen, Trevor Lee, Andrew Pollock, Tao Sun

**Affiliations:** ^1^Center for Precision Medicine, School of Medicine and School of Biomedical Sciences, Huaqiao University, Xiamen, China; ^2^Department of Cell and Developmental Biology, Weill Cornell Medicine, Cornell University, New York, NY, United States; ^3^Department of Neurology, Xiangya Hospital, Central South University, Changsha, China

**Keywords:** Gli3, miR-7, cortical morphogenesis, brain size, neural progenitor

## Abstract

Brain morphogenesis requires precise regulation of multiple genes to control specification of distinct neural progenitors (NPs) and neuronal production. Dysregulation of these genes results in severe brain malformation such as macrocephaly and microcephaly. Despite studies of the effect of individual pathogenic genes, the counter-balance between multiple factors in controlling brain size remains unclear. Here we show that cortical deletion of Gli3 results in enlarged brain and folding structures in the cortical midline at the postnatal stage, which is mainly caused by the increased percentage of intermediate progenitors (IPs) and newborn neurons. In addition, dysregulation of neuronal migration also contributes to the folding defects in the cortical midline region. Knockdown of microRNA (miRNA) miR-7 can rescue abnormal brain morphology in *Gli3* knockout mice by recovering progenitor specification, neuronal production and migration through a counter-balance of the Gli3 activity. Moreover, miR-7 likely exerts its function through silencing target gene Pax6. Our results indicate that proper brain morphogenesis is an outcome of interactive regulations of multiple molecules such as Gli3 and miR-7. Because miRNAs are easy to synthesize and deliver, miR-7 could be a potential therapeutic means to macrocephaly caused by Gli3-deficiency.

## Introduction

Development of the cerebral cortex requires expansion of distinct neural progenitors (NPs) in the ventricular zone (VZ) and subventricular zone (SVZ) and specification of newborn neurons in the intermediate zone (IZ) and cortical plate (CP; Kriegstein et al., 2006; Molyneaux et al., 2007; Molnar, 2011; Franco and Müller, 2013; Ostrem et al., 2014). The precise regulation of specific transcription factors and related signal pathways is critical for proper gene expression, and in turn proliferation, specification and differentiation of NPs (Aguirre et al., 2010; Delaunay et al., 2017). Dysregulation of gene expression results in various brain malformations, such as macrocephaly, which refers to an enlarged brain and microcephaly, which means a smaller brain (Chenn and Walsh, 2002; Ljungberg et al., 2009; Pollock et al., 2014; Sun and Hevner, 2014; Doobin et al., 2016; Shimada et al., 2016). Despite focusing on functional studies of correlatively pathogenic genes, less research has been done to investigate possibilities of their counter-regulatory genes as potential modulators of brain morphogenesis and therapeutic targets of malformation.

Studies have shown that mutations or dysregulations of diverse genes such as *RAB39B*, X-linked *PAK3* and *GRIN2B* can cause macrocephaly in humans (Morisada et al., 2016; Hertecant et al., 2017; Woodbury-Smith et al., 2017). *PTEN* mutation has been shown to be associated with macrocephaly and autism syndrome (Klein et al., 2013; Huang et al., 2016; Kurata et al., 2018). Gli3 has also been identified as a pivotal factor related to severe brain malformation, including macrocephaly (Speksnijder et al., 2013; Tanteles et al., 2015). Gli3 is known as a suppressor in the Sonic hedgehog (Shh) pathway and plays critical roles in regulating pattern formation of different tissues and controlling cell fate determination (Ruiz i Altaba, 1997; Brewster et al., 1998; Vestergaard et al., 2005; Blaess et al., 2011; Feijóo et al., 2011; Miyahara et al., 2014). During mammalian neural development, Gli3 has been shown to determine the specification and differentiation of NPs in different regions at different developmental stages (Motoyama et al., 2003; Theil, 2005; Lebel et al., 2007; Kim et al., 2011, 2018; Wang et al., 2011, 2014; Hasenpusch-Theil et al., 2012; Magnani et al., 2013; Petrova et al., 2013). However, how Gli3 interacts with other molecules in controlling brain size is still not clear.

Opposite to macrocephaly, microcephaly is a brain developmental disorder that is mostly caused by aberrant proliferation or survival of NPs (Braun et al., 2017; DiStasio et al., 2017; Moawia et al., 2017; Xu et al., 2017; Chartier et al., 2018; LaConte et al., 2018). Mutations of several genes that control proliferation of NPs such as *CASK*, *ASPM*, *FOXG1* have been identified (Seltzer and Paciorkowski, 2014; Faheem et al., 2015). Besides coding genes, noncoding RNAs are also involved in regulating neural development and developmental neurological disorders (Bian et al., 2013; Sun and Hevner, 2014; Liu and Sun, 2016). MicroRNA (miRNA) miR-7 has been shown to regulate cortical development and protect neurons from apoptosis (Chen et al., 2010; Sanek and Young, 2012; Fragkouli and Doxakis, 2014; Li et al., 2016). Knocking down miR-7 reduces transition of radial glial cells (RGCs) to intermediate progenitors (IPs) and results in microcephaly-like brain defects (Pollock et al., 2014). Whether miR-7, one of the microcephaly-pathogenic genes, could act as a potential modulator to remedy macrocephaly remains obscure.

In this study, we investigate whether the counter-balance interaction between Gli3 and miR-7 is sufficient to correct macrocephaly-like malformation caused by Gli3 deficiency. Conditional knockout of *Gli3* causes a significant larger brain size, which can be recovered by knockdown of miR-7. Cortical midline folding in *Gli3* knockout brains can be rescued by silencing miR-7 through an opposite effect of Gli3 in specification of NPs and neuronal production. Moreover, we show that Pax6 is a target of miR-7, implying a potential candidate conducting the counteractive role of miR-7 with Gli3. Our results indicate that interactions between Gli3 and miR-7 play a crucial role in controlling brain size and proper cortical morphogenesis. Moreover, targeting miR-7 could offer a potential therapeutic way to Gli3-deficiency induced macrocephaly.

## Materials and Methods

### Transgenic Mice

Generation of floxed *Gli3* transgenic mice (*Gli3*^fl/fl^, exon eight flanked by *loxP* sites) were described by Blaess et al. (2008). Deletion of *Gli3* was generated using the *Emx1-Cre* line (Gorski et al., 2002). Mutant mice with different genotypes were achieved by following the interbreeding strategy in Supplementary Figure S1. Briefly, *Gli3*^fl/fl^ mice were mated with heterozygous *Emx1-Cre*;*Gli3*^fl/+^ mice to obtain homozygous *Emx1-Cre*;*Gli3*^fl/fl^ mice (EG), and EG-type mice were mated with *miR-7*-Sponge containing mice (ES; Pollock et al., 2014) to generate heterozygous *Emx1-Cre*;*Gli3*^fl/+^ mice carrying *miR-7-Sponge* (*Emx1-Cre*;*Gli3*^fl/+^;*miR-7-SP*); and this offspring was further interbred into homozygous mice *Emx1-Cre*;*Gli3*^fl/fl^;*miR-7*-Sponge (EGS) using homozygous *Gli3*^fl/fl^ mice. *Emx1-Cre*;*Gli3*^+/+^ or *Emx1-Cre*;*Gli3*^fl/+^ mice were used as controls. Embryonic day 0.5 (E0.5) was counted from midday of the day of vaginal plug discovery. For each marker and stage, at least three embryos were analyzed. All procedures and protocols were approved by the Institutional Animal Care and Use Committee of the Weill Cornell Medical College (#2011-0062) and conducted in accordance with the National Institutes of Health Guide for the Care and Use of Laboratory Animal (NIH publications Nos. 80-23, revised 1996).

### Tissue Preparation and Nissl Staining

Brain tissues were collected and fixed in 4% paraformaldehyde (PFA) in phosphate-buffered saline (PBS) overnight and subsequently incubated in 25–30% sucrose in PBS, embedded in OCT and stored at –80°C until use. Brains tissues were coronally sectioned into 14–16 μm slides using a cryostat. Sections were washed and stained with 0.5% thionine for 10 min at 50°C followed by 15-min differentiation in 95% ethyl alcohol. The sections were then dehydrated twice in 100% alcohol for 5 min and cleared in xylene for another 5 min twice. Finally, all sections were mounted by resinous medium.

### Immunohistochemistry

To label proliferative neural progenitor cells (NPCs) in the developing cortex, one dose of BrdU (50 μg/g body weight) was administered by intraperitoneal (I.P.) injection in mice at 1 h before sacrifice. Brain tissue sections for each genotype were collected as described above. Before antigens were recovered, sections were proceeded for 15-min incubation in heated (95–100°C) antigen recovery solution (1 mM EDTA, 5 mM Tris, pH 8.0) and 20-min cooling treatment at 4°C. After 1-h blocking in 10% normal goat serum (NGS) in PBS with 0.1% Tween-20 (PBT), sections were incubated with primary antibodies at 4°C overnight, then visualized after 1.5-h of co-culturing with goat anti-rabbit IgG–Alexa- Fluor-488 and/or goat anti-mouse IgG–Alexa-Fluor-546 (1:300, Molecular Probes) at room temperature. Images were captured using a Leica digital camera under a fluorescent microscope (Leica DMI6000B) or a Zeiss confocal microscope.

Primary antibodies against the following antigens were used: bromodeoxyuridine (BrdU; 1:50, Developmental Studies Hybridoma Bank at University of Iowa (DSHB)), Pax6 (1:500, Covance), Pax6 (1:15 DSHB), Tbr1 (1:500, Abcam), Tbr2 (1:500, Abcam) and Satb2 (1:1,000, Abcam).

### Cell Counting

For cell counting from the coronal brain sections of the cortical midline, cells were counted on a fixed width (190 μm bin) of a representative column in the midline of embryonic day 15.5 (E15.5) brains and in a fixed area (300 μm × 430 μm) in the midline of P0 brains. For cell counting from the coronal brain sections of the cerebral cortex, cells were counted on a fixed width (190 μm bin) of a representative column in the cortical wall. All sections analyzed were selected from a similar medial point on the anterior-posterior cortical axis. Cell counting was performed in minimal three chosen areas from at least three sections for each brain, and at least three brains were analyzed in each group. Cell counting in each chosen area was repeated three times and a mean was obtained.

For Satb2^+^ cell counting in the upper layers, the upper region was distinguished from deeper region by dotted lines in each figure according to expression of Tbr1^+^ marker, which is reported to mark deeper layers (Kolk et al., 2006).

### Migration Analysis

The measurement of neuronal migration was descripted by Nowakowski (Nowakowski et al., 2013) with moderate modification. Briefly, each cortical region in a brain section was equally divided into 10 bins from the top of cortical surface to the bottom of ventricle base. The division of each layer was extended along the undulated cortical surface. Tbr1^+^/DAPI^+^ cells or Satb2^+^/DAPI^+^ cells in each bin was counted. Migration was assessed by comparing the ratio of counted cells in each bin vs. whole bins. Because of the folding structure in the midline region of the EG brain, the cell counting area for each brain was limited in a box. To precisely compare migration, we performed the distributive percentage of marked cells in each bin vs. all counting cells.

### Luciferase Assays

For the luciferase reporter constructs, DNA fragments encoding the mouse *Pax6* 3′ untranslated region (3′UTR) were amplified using the following primers: 3′UTR-F, 5′-ACGACTAGTAGCATGTGATCGAGAGAGGAA-3′; 3′UTR-R, 5′-GTGAACAACTGCAAAAC ACTTAGG-3′. DNA fragments were subcloned into a pGL4.13 Luciferase vector (Promega).

To amplify mouse miR-7a-2 precursor, the following primers were used: miR-7a-2-F, 5′-TACAGGAGTGTC CGGCTGAT-3′; miR-7a-2-R, 5′-CAAAATCACTAGTCTTCCAA ACG-3′. To generate miR-7a-2 mutations, the following primers were used: miR-7-mut-F, 5′-CCAACAACAAGTCCCACTGTGGCACATGGTGCT GGTCA-3′; miR-7-mut-R, 5′-TGACCAGCACCATGTGCCACAGTGGGACTT GTTGTTGG-3′.

To generate miR-7 sponge and its mutation, the following primers were used: miR-7-sponge-F, 5′-GCTAACTAGTACAACAAAATCAGGGTCTTCCAGTTATCACAACAAAATCAGGGTCTTCCAG TTATCACAACAAAATCAGGGTCTTCCATCTAGAGATC-3′; miR-7-sponge-R, 5′-GATCTCTAGATGGAAGACCCTGATTTTGTTGTGATAACTGGAAGACCCTG AT TTTGTTGTGATAACTGGAAGACCCTGATTTTGTTGTACTAGTTA GC-3′. miR-7-sponge-mut-F, 5′-GCTAACTAGTACAACAAAATCAGGCTGTTGCAGTTATC ACAACAAAATCAGGCTGT TGCAGTTATCACAACAAA ATCAGGCTGTTGCATCTAGAGATC-3′; miR-7-sponge-mut-R, 5′-GATCTCTAGATGCAACAGCCTGA TTTTGTTGTGATAACTGCAACAGCCTGATTTTGTTGTGATAACTGCAACAGCCTGATTTTGTTGTACTAGTTA GC-3′.

All the luciferase assays were conducted in Neuro2a cells using Lipofectamine 2,000 (Invitrogen) for plasmid transfection, following the manufacturer’s protocol. Plasmids were quantified by UV spectrophotometry and used for transfection in a 2:1 ratio (miRNA: target luciferase constructs). pGL4.13 firefly luciferase was used for the 3′UTRs of targets. pGL4.73 *Renilla* luciferase (Promega) was used as a transfection control.

For transfections, Neuro2a were diluted in DMEM and plated into 24-well plates in triplicate at 1.5 × 10^4^ cells/100 μl. Adherent cells were co-transfected with 100 ng/mL of luciferase reporter containing the *Pax6–*3′UTR and 50nM of either pcDNA3.1 only (control), miR-7a-2 or miR-7a-2-mut, and with pcDNA-iCre, miR-7a-2-SP, or miR-7a-2-sponge mutation (SPmut), respectively. After 48 h, luciferase was measured using the Dual-Luciferase Reporter Assay kit (Promega) using the manufacturer’s protocol and detected on a Victor3 1420 multilabel counter (Perkin Elmer). All conditions were ran in triplicates, and all experiments were repeated at least three times with similar results. Raw data for each condition were normalized for transfection efficiency as were the ratio of *Firefly* luciferase to *Renilla* luciferase. Finally, for each luciferase tested, the empty vector control experiment was set to 1for display.

### Statistics

All data were shown as mean ± SEMT. One-way analysis of variance (ANOVA) with *post hoc* contrasts were used for statistical analysis. The results were considered significant at a probability of less than 0.05.

## Results

### Opposite Effect of Gli3 and miR-7 on Regulation of Brain Size

**Figure 1:**
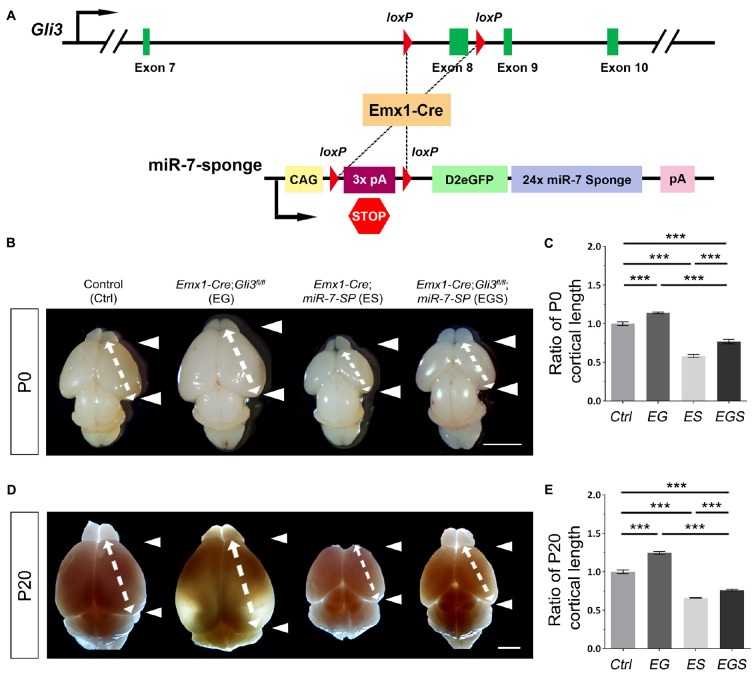
Gli3-deficiency induces brain malformation, which is rescued by depletion of miR-7. **(A)** The genetic modification in *Emx1-Cre*;*Gli3*^fl/fl^; miR-7-Sponge (EGS) mice model was induced by *Emx1-Cre* recombinase-dependent loss of floxed Exon8 in the *Gli3* gene, and an expression of miR-7 sponge. **(B–E)** Cortical loss of Gli3 resulted in a notably larger and longer cortex, while the absence of miR-7 had the opposite effect of Gli3 at both P0 and P20. Values represent mean ± SEM. *n* > 9. ***P < 0.001. One-way ANOVA with *post hoc* test was used. Scale bar = 5 mm.

Previous reports have shown that Gli3 deficiency results in an enlarged brain (Speksnijder et al., 2013; Tanteles et al., 2015). To confirm the role of Gli3 in regulating brain size, floxed *Gli3* transgenic mice were bred with *Emx1-Cre* mice to generate *Gli3* conditional knockout (cKO) mice, in which *Gli3* is deleted only in the cerebral cortex due to the cortical specific activity of Emx1 (Figure 1A; Gorski et al., 2002). As expected, the cortex of *Gli3* knockout mice (*Emx1-Cre*;*Gli3*^fl/fl^, named EG mice) was significantly enlarged at postnatal day 0 (P0) and P20, compared to control mice (see “Materials and Methods” section for grouping of control mice; Figures 1B,D). In addition, our previous study has reported that knockdown of miR-7 using miR-7-specific sponge causes a decrease in the size of the cortex (Pollock et al., 2014). Floxed miR-7 sponge mice (*miR-7-SP*) were also bred with *Emx1-Cre* mice to specifically knock down miR-7 in the cortex, called *Emx1-Cre*;*miR-7-SP*, or ES mice (Figure 1A).

The opposite brain morphologies of EG and ES mice made us speculate that Gli3 and miR-7 might play counter-balancing roles in controlling brain size. To test this possibility, *Gli3* knockout mice (*Emx1-Cre*;*Gli3*^fl/fl^) were bred with *miR-7-SP* containing mice to generate heterozygous *Emx1-Cre*;*Gli3*^fl/+^;*miR-7-SP* mice, which were subsequently bred with homozygous *Gli3*^fl/fl^ mice to generate homozygous *Gli3-miR-7*-double-deficient mice (*Emx1-Cre*;*Gli3*^fl/fl^;*miR-7*, called EGS mice; Supplementary Figure S1). In this strategy, *Emx1-Cre* would induce functional loss of Gli3 and simultaneous silence of miR-7 in cortices of EGS mice. Interestingly, the brain size in EGS mice was significantly smaller than that of EG mice and larger than that of ES mice at P0 and P20, even though it was still smaller than the control group (Figures 1B,D). Moreover, the length of the cerebral cortex was measured (Figures 1C,E). The cortex of EGS mice was longitudinally shorter than that of EG mice and longer than ES mice at P0 and P20 (Figures 1B–E). These results suggest that Gli3 and miR-7 play opposing roles in controlling the brain size.

### Knockdown of miR-7 Rescues Cortical Midline Defects in *Gli3* Knockout Brains

**Figure 2:**
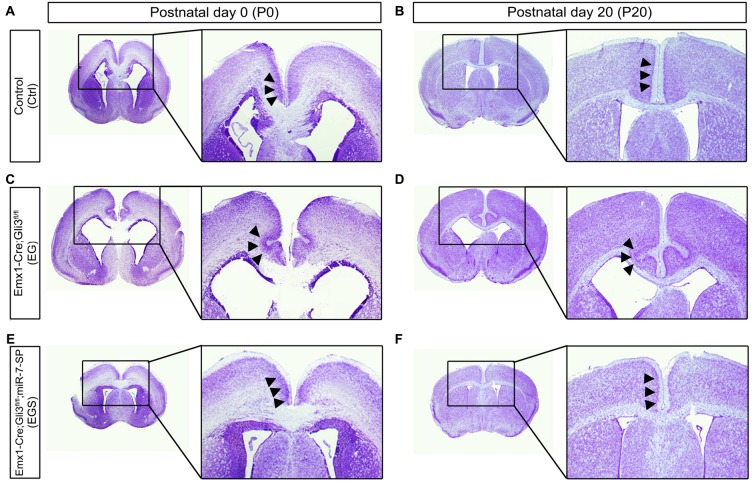
Gli3-deficiency induces abnormal morphogenesis in the cortical midline. **(A,B)** Normal-developing brains displayed smooth cortical surface at the midline of cortices at P0 and P20. **(C,D)** Cortices losing Gli3 showed in-folding phenotype at the midline of cortices at P0, and the defect is maintained at P20. **(E,F)** Conditionally blocking the function of miR-7 from the cerebral cortex corrected the mis-folding defect in the midline in the *Gli3*-deficient brain.

To further examine brain morphology, Nissl staining was performed on sections of control, EG, ES and EGS brains at P0 and P20 (Figure 2, Supplementary Figure S2). The ventricle of EG brains was noticeably larger than the ventricle of control and EGS brains (Figure 2). In P0 brains, the thickness of cortices did not show significant changes among the control, EG and EGS brains, while the ES brains did (Supplementary Figure S2). These results suggest that the difference of brain size, regulated by Gli3 and miR-7, might be due to changes in the ventricle size but not cortical thickness.

Noticeably, folding structures were detected in the cortical midline region in EG brains at P0 and P20 (Figures 2C,D). Instead of forming a smooth cortical surface in the control brain, the CP invaginated into the intermediate zone in the EG cortex (Figures 2C,D). Interestingly, the folding defects in the EG brain were completely recovered in the EGS cortex (Figures 2E,F). The midline folding defects were observed in all *Gli3*-deficient mice (>10 animals), and these defects were rescued in all *Gli3-miR-7*-double-deficient mice (>10 animals). Our results suggest that knockdown of miR-7 is sufficient to rescue midline morphogenesis defects caused by loss of Gli3.

### Knockdown of miR-7 Partially Corrects Abnormal Progenitor Development in the Cortical Midline of *Gli3* Knockout Brains

**Figure 3:**
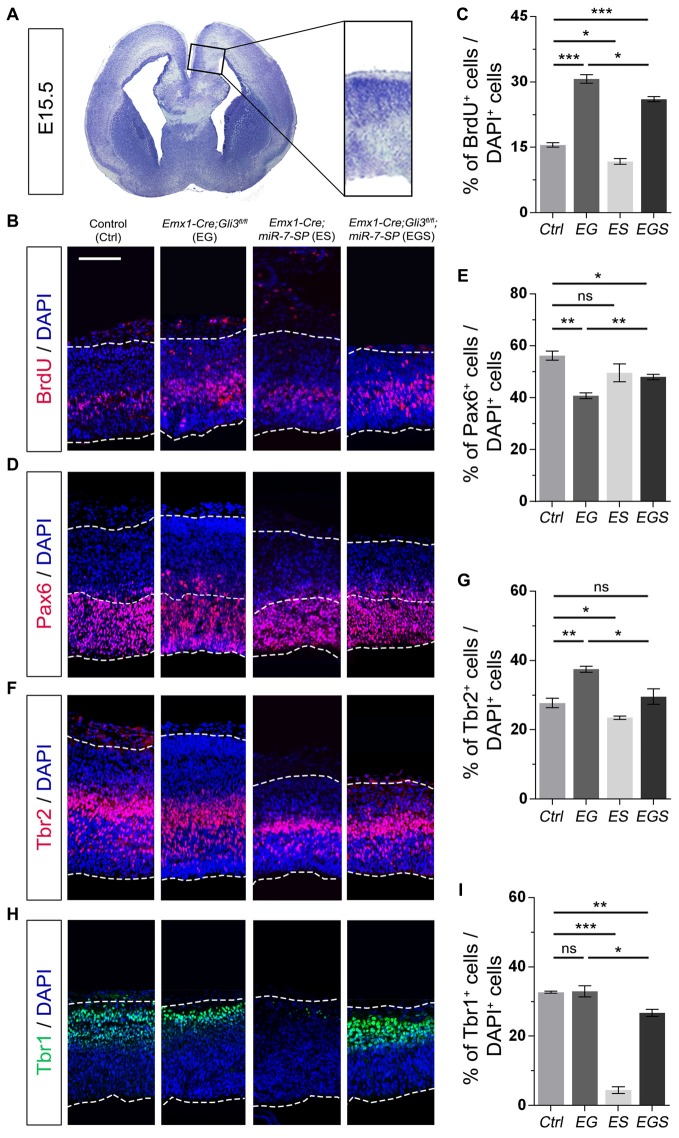
Gli3 effects on proliferation of neural progenitors (NPs) in the cortical midline. **(A)** The position that was measured for the embryonic day 15.5 (E15.5) midline cortex. **(B,C)** Cortical deficiency of Gli3 increased the proportion of BrdU^+^ cells vs. DAPI^+^ cells. Knockdown of miR-7 decreased the proportion of BrdU^+^ cells vs. DAPI^+^ cells. Silencing miR-7 failed to rescue the dysregulation of BrdU^+^ cells in the *Gli3*-deficient brain. **(D,E)** Cortical deficiency of Gli3 suppressed the population of cells expressing the radial glial cell (RGC) marker Pax6^+^/DAPI^+^, which was completely rescued by silencing miR-7. Silencing miR-7 showed no significant changes of the proportion of cells expressing RGC marker Pax6^+^/DAPI^+^. **(F,G)** Losing Gli3 increased the ratio of intermediate progenitor (IP) marker Tbr2^+^/DAPI^+^, which was restored by blocking miR-7. Knockdown of miR-7 did not affect the ratio of IP marker Tbr2^+^/DAPI^+^ at E15.5. **(H,I)** No difference was observed in Tbr1^+^ cells vs. DAPI^+^ cells in EG mice, which was significantly reduced by silencing miR-7. Cortical deficiency of miR-7 decreased the proportion of Tbr1^+^ cells vs. DAPI^+^ cells. The markers BrdU, Pax6, Tbr2, Tbr1 and DAPI stained for proliferative NPs, RGCs, IPs, newborn neurons and all cells, respectively. Values represent mean ± SEM. *n* > 9. *P < 0.05; **P < 0.01; ***P < 0.001; ns, not significant. One-way ANOVA with *post hoc* test was used. Scale bar = 100 μm.

To investigate why Gli3 deficiency caused cortical midline folding, we examined progenitor development in the midline in cortices at E15.5 (Figure 3A). BrdU pulse was introduced to label dividing cells in the S-phase in a cell cycle. Functional inactivation of *Gli3* in EG mice significantly increased the percentage of BrdU^+^ cells vs. DAPI^+^ cells in the cortical midline region, suggesting that losing Gli3 facilitates proliferation of NPs (Figures 3B,C). Furthermore, we analyzed populations of RGCs, IPs and newborn neurons, by labeling them with Pax6, Tbr2 and Tbr1, respectively. The proportion of Pax6^+^ cells vs. DAPI^+^ cells was decreased in the cortical midline of EG brains, suggesting that loss of Gli3 reduces the population of RGCs (Figures 3D,E). However, the population of basally dividing Pax6^+^ progenitors among all Pax6^+^ cells, and Tbr2^+^ IPs vs. DAPI^+^ cells were both increased in the *Gli3*-deficient cortical midline (Figures 3F,G; Supplementary Figure S3A). In addition, no detectable changes were found in the population of Tbr1-marked newborn neurons in the EG cortex (Figures 3H,I). Our results suggest that loss of Gli3 might lead to elevated basally dividing Pax6^+^ progenitors and increased transition from RGCs to IPs in the cortical midline region.

Moreover, the percentage of BrdU^+^/DAPI^+^ cells was reduced in the cortical midline region in E15.5 ES brains, but increased in EG brains, compared to controls, suggesting an opposite role of miR-7 to Gli3 in controlling proliferative process of NPs (Figures 3B,C). The population of Tbr2^+^ IPs vs. DAPI^+^ cells were slightly decreased in ES mice (Figures 3F,G). The population of Tbr1-marked newborn neurons was specifically decreased in ES mice (Figures 3H,I). No detectable changes were found in the population of Pax6^+^ RGCs vs. DAPI^+^ cells (Figures 3D,E).

Next, we examined progenitor development in the cortical midline in EGS mice. The population of cycling NPs that took up BrdU in the cortical midline region in EGS mice was still higher than that in the control group, but lower than that in the EG mice (Figures 3B,C). Moreover, knockdown of miR-7 partially restored the decreased population of Pax6^+^ RGSs and completely reversed the increased percentage of the basally dividing Pax6^+^ progenitors and Tbr2^+^ IPs in the midline of *Gli3* deficient brains (Figures 3D–G; Supplementary Figure S3A). Furthermore, knockdown of miR-7 specifically reduced the population of Tbr1-marked newborn neurons in the *Gli3*-deficient cortical midline (Figures 3H,I). These data indicate that blocking miR-7 rescues midline folding in *Gli3*-deficient cortices by possibly correcting the number of the basally dividing Pax6^+^ progenitors, the transition of RGCs to IPs and in turn, and the production of newborn neurons.

### Opposite Roles of Gli3 and miR-7 in Controlling Neuronal Production

**Figure 4:**
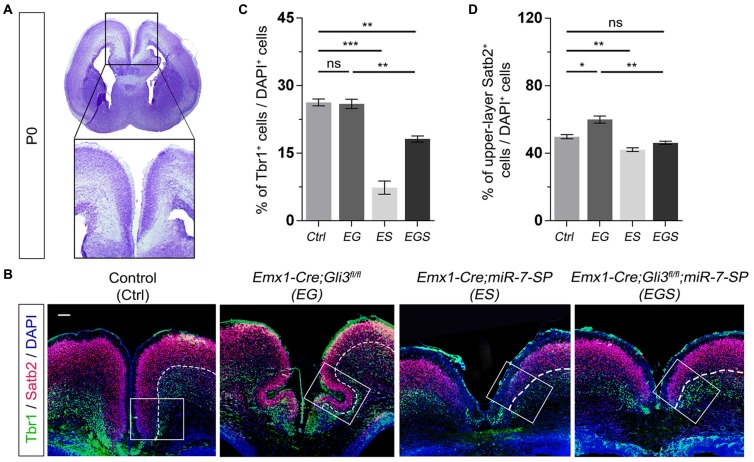
Gli3 and miR-7 regulate population of newborn neurons in the cortical midline through an opposite effect. **(A)** The position that was captured and presented in the P0 cortical midline region. **(B,C)** Absence of Gli3 showed no alteration of Tbr1^+^/DAPI^+^ neurons in the deep layer. But losing function of both miR-7 and Gli3 significantly reduced the population of Tbr1^+^/DAPI^+^ cells. Knockdown of miR-7 significantly reduced the proportion of Tbr1^+^/DAPI^+^ neurons in the deep layer.** (B,D)** The upper layer neurons were separated according to the zones of Satb2^+^ intensive cell layer and Tbr1^+^ marked layer using the white dotted line. Cortical deficiency of Gli3 increased the proportion of upper layer Satb2^+^/DAPI^+^ cells, which was rescued by silencing miR-7. Knockdown of miR-7 significantly reduced the proportion of Satb2^+^/DAPI^+^ neurons in the upper-layer. The markers Tbr1, Satb2 and DAPI stained for newborn neurons in deeper layer, newborn neurons in upper layer and all cells, respectively. Values represent mean ± SEM. *n* > 9. *P < 0.05; **P < 0.01; ***P < 0.001; ns, not significant. One-way ANOVA with *post hoc* test was used. Scale bar = 100 μm.

To further examine the outcome of altered neural progenitor development in the cortical midline, we analyzed neuronal production in P0 EG and EGS brains (Figure 4A). Previous studies have demonstrated that Tbr1 is mostly expressed in deep layer neurons and Satb2 is highly expressed in upper layer neurons of the cerebral cortex (Britanova et al., 2005; Kolk et al., 2006; Szemes et al., 2006). There was no detectable difference in the percentage of Tbr1^+^ cells in the EG cortex, suggesting that generation of deep layer neurons is not affected by loss of *Gli3* (Figures 4B,C). However, the percentage of Satb2 marked upper layer cells was increased compared to controls, indicating an elevated production of upper layer neurons in the cortical midline of *Gli3* knockout brains (Figures 4B,D). In addition, both deep layer Tbr1^+^ cells and upper layer Satb2^+^ cells were significantly reduced in the ES cortex (Figures 4B–D).

Moreover, knockdown of miR-7 in *Gli3* knockout brains caused a decrease in the percentage of Tbr1^+^ and Satb2^+^ upper layer cells, which indicates that knockdown of miR-7 partially rescues elevated production of upper layer neurons caused by *Gli3* deletion (Figures 4B–D).

### Gli3 and miR-7 Function Oppositely on Cerebral Cortical Region

**Figure 5:**
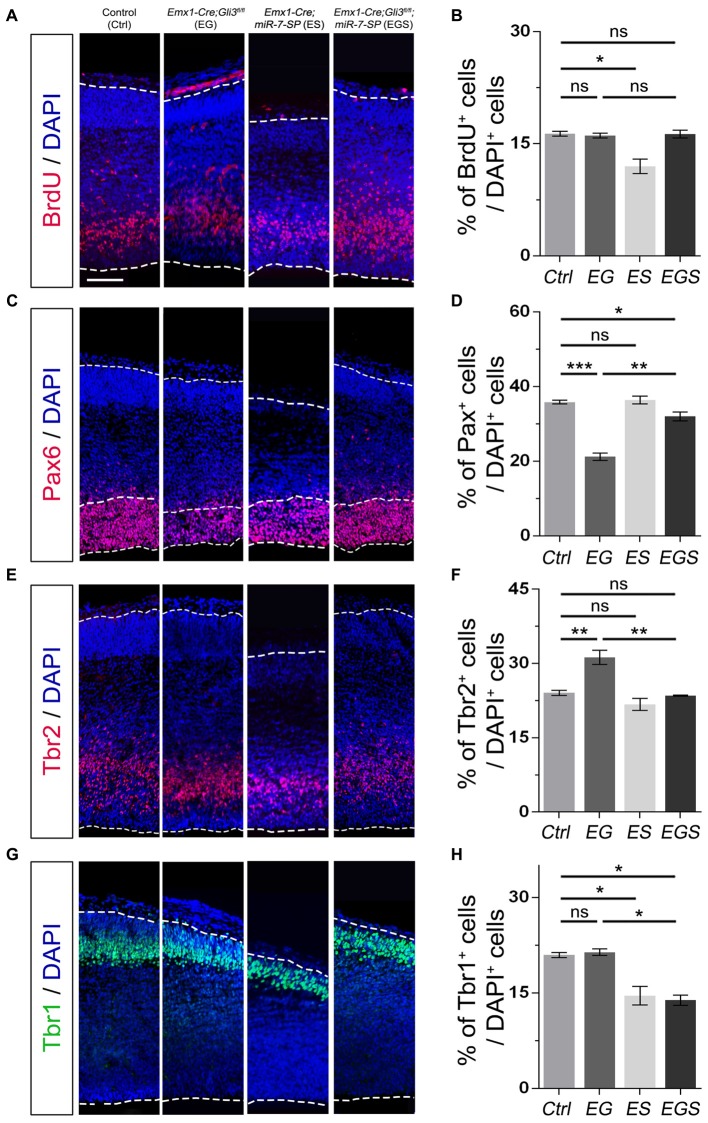
Gli3 and miR-7 effects on the proliferation of NPs in the cerebral cortex. **(A,B)** Cortical deficiency of Gli3 did not have an impact on the proportion of BrdU^+^ cells vs. DAPI^+^ cells. Cortical deficiency of miR-7 decreased the proportion of BrdU^+^ cells vs. DAPI^+^ cells. **(C,D)** Cortical deficiency of Gli3 suppressed the population of cells expressing RGC marker Pax6^+^/DAPI^+^, which was partially rescued by silencing miR-7. Silencing miR-7 showed no significant change of the proportion of cells expressing RGC marker Pax6^+^/DAPI^+^ at E15.5. **(E,F)** Losing Gli3 increased the ratio of IP marker Tbr2^+^/DAPI^+^, which was restored by blocking miR-7. Knockdown of miR-7 did not affect the ratio of IP marker Tbr2^+^/DAPI^+^. **(G,H)** No difference was observed in Tbr1^+^ cells vs. DAPI^+^ cells in EG mice, which was significantly reduced by silencing miR-7. Cortical deficiency of miR-7 decreased the proportion of Tbr1^+^ cells vs. DAPI^+^ cells. The markers BrdU, Pax6, Tbr2, Tbr1 and DAPI stained for proliferative NPs, RGCs, IPs, newborn neurons and all cells, respectively. Values represent mean ± SEM. *n* > 9. *P < 0.05; **P < 0.01; ***P < 0.001; ns, not significant. One-way ANOVA with *post hoc* test was used. Scale bar = 100 μm.

To analyze whether Gli3 and miR-7 also function oppositely outside the cortical midline, we examined the cerebral cortical region of E15.5 EG, ES and EGS brains (Figure 5). We found no detectable difference in the percentage of BrdU^+^ cells between control and EG mice (Figures 5A,B). However, we discovered that losing Gli3 reduces the percentage of Pax6^+^ cells but increases the percentage of Tbr2^+^ IPs in the cerebral cortex of *Gli3* knockout brains (Figures 5C–F). These data suggest that losing Gli3 reduces the population of RGCs but expands the population of IPs in the cerebral cortical region. Similarly, *Gli3*-deficiency did not alter the percentage of Tbr1^+^ cells, indicating that Gli3 had no effect on Tbr1 marked newborn neurons in the EG cortical region (Figures 5G,H). Moreover, we also observed slight reductions of BrdU^+^ cells and Tbr1^+^ cells vs. DAPI^+^ cells in the ES cortex (Figures 5A,B,G,H), suggesting reduced differentiation due to miR-7 knockdown. No significant difference was found in the population of Pax6^+^ RGCs nor Tbr2^+^ IPs in ES mice (Figures 5C–F).

Next, we examined the function of miR-7 in the *Gli3*-deficient cortical region in the EGS cortex. Knockdown of miR-7 had no impact on the percentage of BrdU^+^ cells (Figures 5A,B). The percentage of Pax6^+^ RGCs in the cortical region in EGS mice was higher than that in EG mice, but still slightly lower than that in the control group (Figures 5C,D). Interestingly, the percentages of the basally dividing Pax6^+^ progenitors among all Pax6^+^ RGCs were similar among control, EG and EGS (Supplementary Figure S3B). Blocking miR-7 completely reversed the increased amount of Tbr2^+^ IPs detected in EG mice (Figures 5E,F). In addition, knockdown of miR-7 specifically reduced the population of Tbr1-marked newborn neurons in the *Gli3*-deficient cortical region (Figures 5G,H). Our results indicate that expression of Gli3 is required for determining the fate and differentiation of NPs, which is oppositely regulated by miR-7 during cerebral cortical development.

Moreover, the outcome of altered neural progenitor development in the cortical region was analyzed via neuronal production in P0 EG, ES and EGS brains (Supplementary Figure S4A). While there was a reduction in the percentage of Tbr1^+^ cells and Satb2^+^ cells in the ES cortex, no detectable difference was measured in the EG cortex, suggesting that generation of both deep and upper layer neurons is not affected by the loss of *Gli3* (Supplementary Figure S4). Knockdown of miR-7 in *Gli3* knockout brains caused a decreased percentage of Tbr1^+^ but not Satb2^+^ cells, which indicates that knockdown of miR-7 affects the development of deep layer neurons (Supplementary Figure S4).

### Neuronal Migration in the Midline Region Is Distinctly Regulated by Gli3 and miR-7

**Figure 6:**
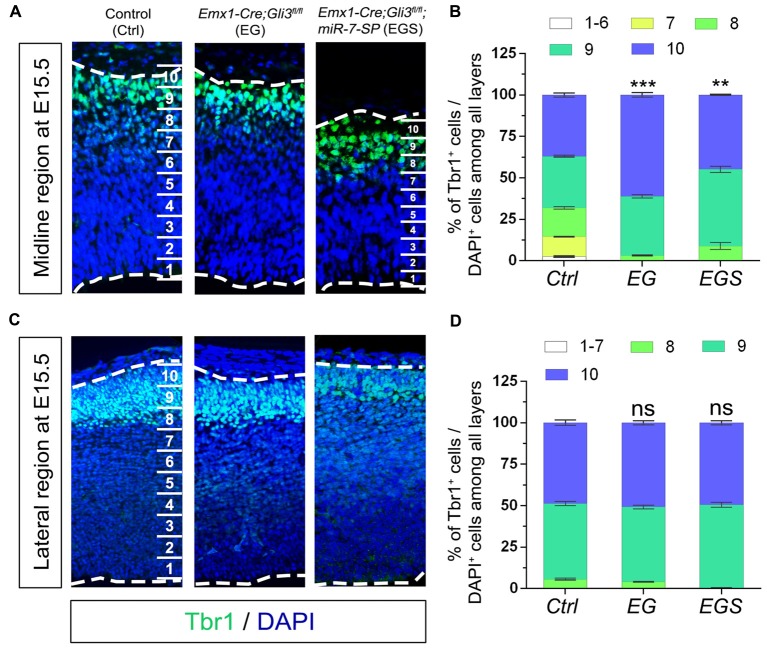
The regulatory role of Gli3 and miR-7 in neuronal migration in E15.5 cortices. **(A,B)** Absence of Gli3 facilitated more Tbr1^+^ marked neurons to migrate into the upper-bins (bin10) in the midline region, while double-lose of miR-7 and Gli3 corrected the migratory process. **(C,D)** No significant alteration of migratory process was detected in the bin10 in the lateral region in EG and EGS cortices. The markers BrdU, Tbr1 and DAPI stained for proliferative NPs, newborn neurons and all cells, respectively. Values represent mean ± SEM. *n* > 9. **P < 0.01; ***P < 0.001; ns, not significant. One-way ANOVA with *post hoc* test was used. Scale bar = 50 μm.

Because neural progenitor development and neuronal production are altered by loss of Gli3, we speculated that neuronal migration also might be affected. To measure migration, the midline and lateral cortical regions in a brain section were equally divided into 10 bins from the top of cortical surface (as bin10) to the bottom of ventricle base (as bin1). We first compared distribution of Tbr1^+^/DAPI^+^ cells in the midline and lateral regions of E15.5 control, EG and EGS brains (Figure 6). Fewer newborn neurons stayed into bin1–7 and more migrated into bin10 in the midline region of *Gli3* knockout brains, compared to those in controls (Figures 6A,B). However, knockdown of miR-7 in *Gli3* knockout cortex specifically corrected the migratory process of newborn neurons in bin10 (Figures 6A,B). In addition, except fewer neurons detected in bin8 in the EGS cortex, no significant difference was observed in bin10 in the cortical lateral region in control, EG and EGS brains (Figures 6C,D). These data suggest that more newborn neurons migrate into the most upper bins in the cortical midline region of Gli3 deficient brains.

**Figure 7:**
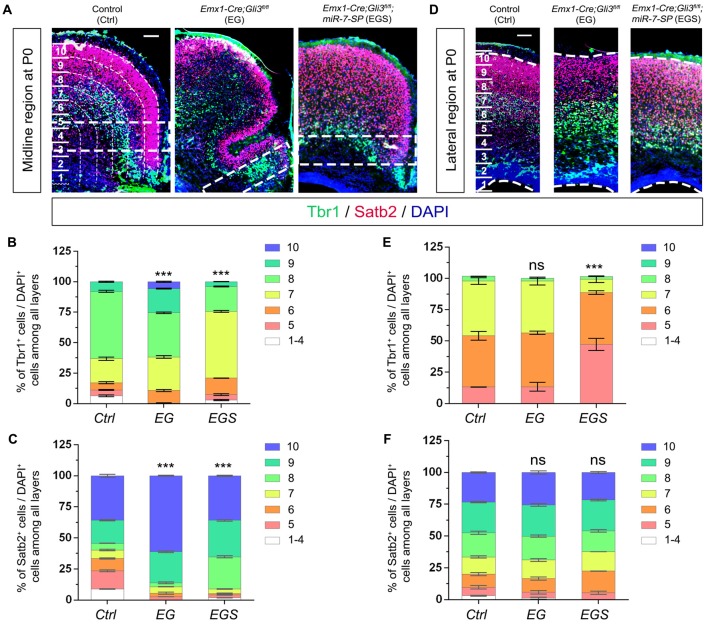
The regulatory role of Gli3 and miR-7 in modulating neuronal migration in the P0 cortex. **(A–C)** Absence of Gli3 facilitated more Tbr1^+^ and Satb2^+^ marked neurons to migrate into the upper bins in the midline region, while double-lose of miR-7 and Gli3 partially corrected the migratory process. **(D–F)** Subtle alteration of migratory process was detected in the upper bins in the cortical lateral region in EG and EGS brains. The markers Tbr1, Satb2 and DAPI stained for deeper bin newborn neurons, upper-bin newborn neurons and all cells, respectively. Values represent mean ± SEM. *n* > 9. ***P < 0.001; ns, not significant. One-way ANOVA with *post hoc* test was used. Scale bar = 100 μm.

We next evaluated migration of newborn neurons in P0 cortices by counting the percentage of Tbr1^+^/DAPI^+^ cells and Satb2^+^/DAPI^+^ cells (Figure 7). More newborn neurons migrated towards the upper bins (bin9 and 10) in the midline region of *Gli3*-deficient brains (Figures 7A–C). The preferred upper bin migration of newborn neurons in the midline region of *Gli3*-deficient brains was significantly corrected by knockdown of miR-7 in EGS brains (Figures 7A–C). No significant migratory alteration was detected in the cortical lateral region in control, EG and EGS brains, except that fewer Tbr1^+^ cells were detected in bin5 and 7 in EGS brains (Figures 7D–F). These results indicate that miR-7 knockdown can rescue exceeded neuronal migration that is detected only in the midline but not lateral region in *Gli3*-deficient brains.

### Gli3 and miR-7 Causes Distinct Degrees of Changes of Neural Development in the Cortical Midline and Lateral Regions

To further analyze the reasoning of the limited folding structure in the midline region in *Gli3*-deficient brains, we evaluated the degree of changes of neural progenitor proliferation and neuronal production by comparing the midline and lateral regions in EG and EGS brains (Supplementary Figure S5). In EG brains, higher fold changes of BrdU^+^, Pax6^+^ and Tbr2^+^ cells at E15.5, and Satb2^+^ cells at P0 were detected in the midline region than the lateral (Supplementary Figure S5A,B). Fold changes of Tbr1^+^ cells showed no significant differences between the midline and lateral regions of both E15.5 and P0 brains. These results suggest that more NPs and more upper layer neurons are produced in the midline, compared to those in the lateral region in EG brains.

Moreover, in E15.5 EGS brains, knockdown of miR-7 reduced the higher fold change of BrdU^+^ cells, and corrected those of Pax6^+^ and Tbr2^+^ cells, and increased that of Tbr1^+^ cells, compared between the midline and lateral regions (Supplementary Figure S5C). On the other hand, in P0 EGS brains, knockdown of miR-7 caused reduced fold changes of Tbr1^+^ and Satb2^+^ cells in the midline region (Supplementary Figure S5D). These data suggest that knocking down miR-7 partly rescues abnormal expansion of NPs and newborn neurons in the cortical midline region.

### Pax6 Is Target for miR-7

miRNAs normally function by silencing target genes in the 3′UTR. Potential targets for miR-7 were bioinformatically analyzed using the tool of miRDB^1^. We discovered that the 3′UTR of transcription factor *Pax6* contains a binding site for miR-7a-2 (Supplementary Figure S6A). A bioinformatic RNA structure tool predicted the binding structure and related energetics of Pax6 pairing with miR-7 (Supplementary Figure S6B; Mathews et al., 2004; Lu et al., 2006). Formation of a RNA duplex between *Pax6* 3′UTR and miR-7 required a free energetic level of -13.4 kJ/mol, in which the lower the free energy, the more stable the structure would be. Moreover, there was no complex secondary structure formed using the prediction tool, suggesting that Pax6 is likely a target of miR-7 (Supplementary Figure S6B).

Based on the finding that *Pax6* is required for *Gli3*-mediated telencephalic patterning (Fuccillo et al., 2006b), to further verify miR-7’s targeting effect on Pax6, we performed luciferase assays by testing the *Pax6* 3′UTR (Supplementary Figure S6C). The relative luciferase activity in the construct containing the *Pax6* 3′UTR was significantly repressed by miR-7a-2, but not by miR-7a-2 mutation (miR-7a-2mut; Supplementary Figures S6C,D). miR-7a-2 sponge (miR-7a-2SP) could rescue the repressive effect of miR-7 on the *Pax6* 3′UTR. However, the miR-7a-2 SPmut displayed no capacity to rescue the silencing effect of miR-7a-2 on the *Pax6* 3′UTR (Supplementary Figure S6C). Moreover, the luciferase activity in constructs only containing a blank plasmid (pGL4.13) had no obvious changes in the co-expression of miR-7a-2 and miR-7a-2-SP or miR-7a-2-SPmut (Supplementary Figure S6D). These results indicate that Pax6 is a putative target for miR-7.

## Discussion

Proper cortical development is precisely regulated by diverse genes to ensure the normal architecture and function of the mammalian brain. Mutations of critical genes in brain development can cause severe brain malformation such as macrocephaly and microcephaly. In this study, we analyze the function of two pathogenic genes by breeding *Gli3* knockout mice with miR-7 knockdown mice. We demonstrate that a counter-balance between Gli3 and miR-7 is crucial in controlling brain size and proper morphogenesis by regulating progenitor specification and neuronal production, particularly, in the cortical midline. Our findings imply a potential strategy and possibility to treat *Gli3*-deficiency induced macrocephaly by silencing microcephaly-pathogenic gene miR-7.

Normal cortical morphogenesis relies on the precise generation of RGCs, IPs and newborn neurons (Matsumoto and Osumi, 2008; Rakic, 2009; Aguirre et al., 2010). Expansion of the neural progenitor population is crucial for controlling brain size. During morphogenesis of the cerebral cortex, several structural changes take place when Gli3 is deleted. Loss of Gli3 accelerates the cell cycle of NPs (Wilson et al., 2012). Our results support previous reports that expansion of the neural progenitor pool results in overgrowth of the brain (Zega et al., 2017, 2018). On the other hand, miR-7 knockdown causes a great reduction of proliferative progenitor population and reduced cortical size (Pollock et al., 2014). These observations lead us to test the balanced control of the neural progenitor population by Gli3 and miR-7.

Interestingly, the folding structure is only detected in the cortical midline region in *Gli3*-deficient brains. The following factors might contribute to the midline folding: first, we have found that Gli3 is critical in determining the cell fate of NPs. RGCs normally undergo asymmetric divisions to produce new RGCs for self-renewal, and newborn neurons (Rakic et al., 1974; Homem et al., 2015). Similarly, our study demonstrates a key role for Gli3 in facilitating RGC proliferation while suppressing RGC differentiation. Mis-folding morphogenesis of the cortical midline might result from the dysregulation of cell fate of NPs in *Gli3*-deficient cortex. We observed an elevated expansion of the basally dividing Pax6^+^ progenitors, which are likely the potential Pax6^+^ basal radial glias (bRGs) according to their cortical location. These alterations of potential bRGs enhanced RGC differentiation and IPs proliferation in the *Gli3*-deficient brain causes expansion of newborn neurons in the upper layer, but not the deep layer of the cortical midline, which in turn, elongates the length along cortical surface areas, and eventually results in the invaginated structure in the postnatal cortex. Second, comparison of neural development between the cortical midline and lateral regions also points out that more NPs and newborn neurons are generated in the midline region than lateral region in *Gli3* knockout brains, which might lead to folding structures only in the midline region. Third, our neuronal migration analyses indicate that more newborn neurons migrate into the most upper layers in the midline region than the lateral region of *Gli3*-deficient brain, which might cause an expansion of cortical surface and eventually folding structures.

Moreover, BrdU pulse transiently labels dividing cells in the S-phase of a cell cycle, which reflects progenitors as a whole. Pax6 and Tbr2 label most RGCs and IPs, respectively. In the cortical lateral region, we have found a decrease of Pax6^+^ cells and increase of Tbr2^+^ cells. Unchanged BrdU labeling might reflect a balanced outcome of Pax6^+^ and Tbr2^+^ progenitors, which eventually results in unchanged production of newborn neurons in both deep and upper layers of the cortex. Even though dysregulation of NPs do not cause folding structure in the lateral region, the enlarged brain still physically elongates the lateral cortex in the transverse direction, which might cause unaffected proliferative NPs number in the lateral region.

Gli3 acts as an essential transcription factor regulating the Shh signaling pathway in either an active manner or a repressing manner, due to the two forms of Gli3 (Gli3-activator or Gli3-represser; Fuccillo et al., 2006a). Constitutively active Shh signaling leads to higher proportion of bRGs and IPs, and enlarged brains and midline folding, which is similar to brain defects of *Gli3* knockout mice (Rallu et al., 2002; Wang et al., 2016). Reports from us indicate that Gli3 regulates brain size and midline cortical structure, which is potentially through modulating the Shh signaling as either an activator or a suppressor. Collectively, occurrence of folding structures only in the cortical midline also implies a more profound effect of Gli3 on regulating RGC differentiation and neuronal migration in the midline than in the cortical region.

In this study, we have found that miR-7 plays an opposite role in determining cell fate of NPs, compared to Gli3. Knockdown of miR-7 is sufficient to rescue an enlarged brain size, and cortical midline folding defects in *Gli3*-deficient brains, likely due to the counter-balance effect between Gli3 and miR-7 in regulating the size of the neural progenitor pool, and the transition from RGCs to IPs and newborn neurons (Rallu et al., 2002; Fuccillo et al., 2006a). Considering the defect of *Gli3*-deficient cortex is likely caused by the activation of Shh, the rescuing effect of miR-7 knockdown might be due to the suppression of the Shh signaling. Previous studies have shown that Pax6 antagonizes the Shh activity in a manner similar to Gli3 (Fuccillo et al., 2006b; Matsumoto and Osumi, 2008; Georgala et al., 2011). We and others have found that miR-7 inhibits cell proliferation and origin of forebrain neurons via suppressing its target Pax6 (de Chevigny et al., 2012; Luo et al., 2015). Knockdown of miR-7 might release its suppression effect on Pax6, which may potentially restore telencephalon patterning by blocking increased Shh activity. Correction of the Shh activity due to Gli3 deficiency promotes expansion of RGCs, and rescues the elevated neuronal production in the upper layer in the cortical midline, and eventually corrects midline folding defects. Moreover, beside Pax6, other miR-7 target genes also are likely involved in regulating the Shh signaling pathway, such as Sufu, Fgf family and HoxD family (Chen et al., 2004; Kim et al., 2011, 2018). The future work should be further investigation of specific miR-7 targets that respond to the Shh signaling pathway.

In summary, our study demonstrates that Gli3 and miR-7 play a counter-balancing role in regulating morphogenesis of the postnatal brain by predominantly controlling cell fate of RGCs and production of newborn neurons. Besides miR-7, other microcephaly genes might be involved in restoration of macrocephaly defects, which should be revealed in future studies. Nevertheless, our findings imply that counter-balance between multiple genes such as Gli3 and miR-7 might play a general role in regulating cortical development. This research provides a new perspective of consideration of using pathogenic genes with opposite effects for developing a therapeutic strategy to treat brain malformation.

## Author Contributions

All authors listed have made a substantial, direct and intellectual contribution to the work, and approved it for publication.

## Conflict of Interest Statement

The authors declare that the research was conducted in the absence of any commercial or financial relationships that could be construed as a potential conflict of interest.

## Footnotes

^1^http://mirdb.org/
