# Supplementary material for: Counter-Balance Between Gli3 and miR-7 Is Required for Proper Morphogenesis and Size Control of the Mouse Brain
Source: Front Cell Neurosci. 2018 Aug 17;12:259. doi: 10.3389/fncel.2018.00259 (PMC6121149; doi:10.3389/fncel.2018.00259)
Supplement: Supplementary file 1 [file Data_Sheet_1.PDF]

Counter-balance between Gli3 and miR-7 is required for proper morphogenesis and size control of the mouse brain

Longbin Zhang[1,*], Taufif Mubarak[2,*], Yase Chen[3,*], Trevor Lee[2], Andrew Pollock[2] and Tao Sun[1,2,#]

[1]Center for Precision Medicine, School of Medicine and School of Biomedical Sciences, Huaqiao University, Xiamen, Fujian 361021, China

[2]Department of Cell and Developmental Biology, Cornell University Weill Medical College, 1300 York Avenue, New York, NY 10065

[3]Department of Neurology, Xiangya Hospital, Central South University, Changsha, China

[#]Corresponding author: Dr. Tao Sun, Email: taosun@hqu.edu.cn

*These authors contributed equally to this work.

Running head: Counter-balancing roles between Gli3 and miR-7 in cerebral cortex

Keywords: Gli3, miR-7, cortical morphogenesis, neural progenitor, cilia

SUPPLEMENTARY FIGURE LEGENDS

FIGURE S1. The inbreeding strategy to produce mouse models. The *Gli3*$^{fl/fl}$ mice were mated with *Emx1-Cre*;*Gli3*$^{fl/+}$ mice to obtain *Emx1-Cre*;*Gli3*$^{fl/fl}$ mice (EG), and *EG*-type mice were mated with *miR-7*-Sponge containing mice to obtain *Emx1-Cre*;*Gli3*$^{fl/+}$;*miR-7-SP* mice. Their offspring was further interbred with *Gli3*$^{fl/fl}$ into homozygous mice *Emx1-Cre*;*Gli3*$^{fl/fl}$;*miR-7-Sponge* (EGS). *Emx1-Cre*;*Gli3*$^{+/+}$ or *Emx1-Cre*;*Gli3*$^{fl/+}$ mice were used as controls.

FIGURE S2. No significant effect of Gli3-deficiency on regulating cerebral cortical thickness. (**A,B**) The thickness of outboard cortices showed no disorder in *Gli3*-deficient mice and *Gli3/miR-7*-double-deficient mice, but displayed decreases in *miR-7*-silencing mice. Values represent mean ± SEM. n > 9. ***P < 0.001; ns, not significant. One-way ANOVA with post-hoc test was used.

FIGURE S3. The alteration of basally dividing Pax6^{+} progenitors in E15.5 mice brains. The dotted line in the middle of the cortical wall was used to separated the basally dividing Pax6^{+} progenitors (upper) from all Pax6^{+} marked RGCs. **(A)** Cortical deficiency of Gli3 increased the proportion of Pax6^{+} marked basally dividing progenitors, which was completely rescued by silencing miR-7. Knockdown of miR-7 decreased the proportion. **(B)** Cortical deficiency of Gli3 and miR-7 had no effect on regulating the number of Pax6^{+} marked basally dividing progenitors. Knockdown of

miR-7 decreased the proportion. Values represent mean ± SEM. n > 9. **P < 0.01; ***P < 0.001; ns, not significant. One-way ANOVA with post-hoc test was used. Scale bar = 50 µm.

FIGURE S4. The regulatory role of Gli3 and miR-7 in controlling neuronal production in the cerebral cortex. **(A,B)** Absence of Gli3 showed no alteration of deep layer marker $Tbr1^+/DAPI^+$ cells. But losing function of both miR-7 and Gli3 significantly reduced the proportion of $Tbr1^+/DAPI^+$ cells. Knockdown of miR-7 significantly reduced the proportion of $Tbr1^+/DAPI^+$ neurons in the deep layer. **(A,C)** The upper layer neurons were separated according to the zones of $Satb2^+$ intensive cell layer and $Tbr1^+$ marked layer using the white dotted line. Cortical deficiency of Gli3 and miR-7 had no effect on the proportion of upper layer $Satb2^+$ cells versus $DAPI^+$ cells. Knockdown of miR-7 significantly reduced the proportion of $Satb2^+/DAPI^+$ neurons in the upper-layer. The markers Tbr1, Satb2 and DAPI stained for newborn neurons in deeper layer, newborn neurons in upper layer and all cells, respectively. Values represent mean ± SEM. n > 9. **P < 0.01; ns, not significant. One-way ANOVA with post-hoc test was used. Scale bar = 100 µm.

FIGURE S5. The comparison of neural development in the cortical midline region and lateral region. **(A,B)** The fold changes of $BrdU^+$ cells, $Pax6^+$ cells and $Tbr2^+$ cells in E15.5 cortices, and $Satb2^+$ cells in P0 cortices were higher in the midline than those in the lateral region of EG mice, while $Tbr1^+$ cells showed no difference between two

regions in E15.5 and P0 EG brains. **(C,D)** The fold changes of each marker were decreased by blocking miR-7 in *Gli3*-knockout cortices. Values represent mean ± SEM. n > 9. *P < 0.05; **P < 0.01; ***P < 0.001; ns, not significant. One-way ANOVA with post-hoc test was used.

FIGURE S6. Pax6 is a target of miR-7. **(A)** The *Pax6* 3' untranslated region (3'UTR) contained a binding site for miR-7a-2. The seed sequence is shown in red. **(B)** The predicted binding structure between miR-7a-2 and *Pax6* was simulated. The free energy for stabilization of the structure was -13.4 kJ/mol for double strands of Pax6 and miR-7a-2. **(C)** miR-7a-2 suppressed luciferase activities in the construct containing the *Pax6*-3'UTR, while miR-7a-2-SP, but not miR-7a-2-SPmut, rescued the suppression. miR-7a-2-mut had no suppressing activity. (**D**) None notable changes of luciferase activities were detected in the blank construct. Values represent mean ± SEM. n > 3. *P < 0.05;***P < 0.001; ns, not significant. One-way ANOVA with post-hoc test was used.

FIGURE S1

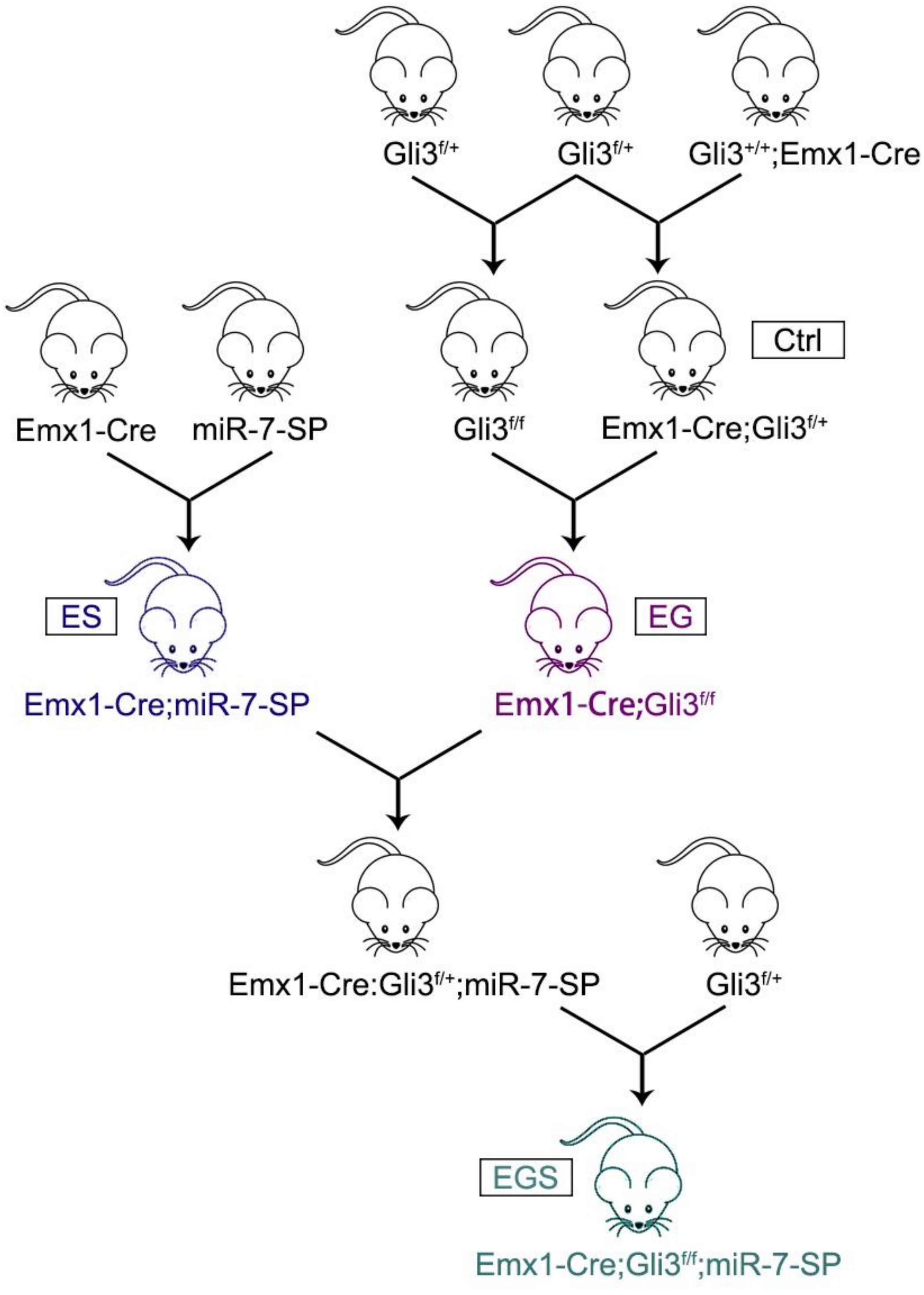
Gli3$^{f/+}$
Gli3$^{f/+}$
Gli3$^{+/+}$;Emx1-Cre
Ctrl
Emx1-Cre
miR-7-SP
Gli3$^{f/f}$
Emx1-Cre;Gli3$^{f/+}$
ES
EG
Emx1-Cre;miR-7-SP
Emx1-Cre;Gli3$^{f/f}$
Emx1-Cre:Gli3$^{f/+}$;miR-7-SP
Gli3$^{f/+}$
EGS
Emx1-Cre;Gli3$^{f/f}$;miR-7-SP

FIGURE S2

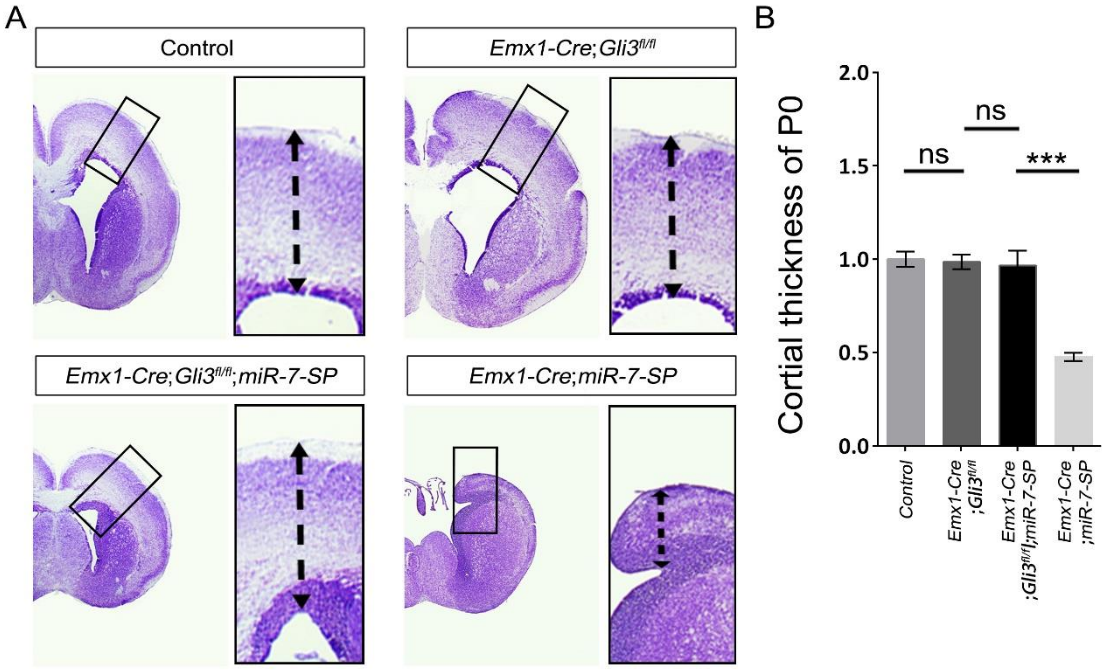
A
Control
Emx1-Cre;Gli3fl/fl
Emx1-Cre;Gli3fl/fl;miR-7-SP
Emx1-Cre;miR-7-SP
B
Cortial thickness of P0
2.0
1.5
1.0
0.5
0.0
ns
ns
***
Control
Emx1-Cre ;Gli3fl/fl
Emx1-Cre ;Gli3fl/fl;miR-7-SP
Emx1-Cre ;miR-7-SP

FIGURE S3

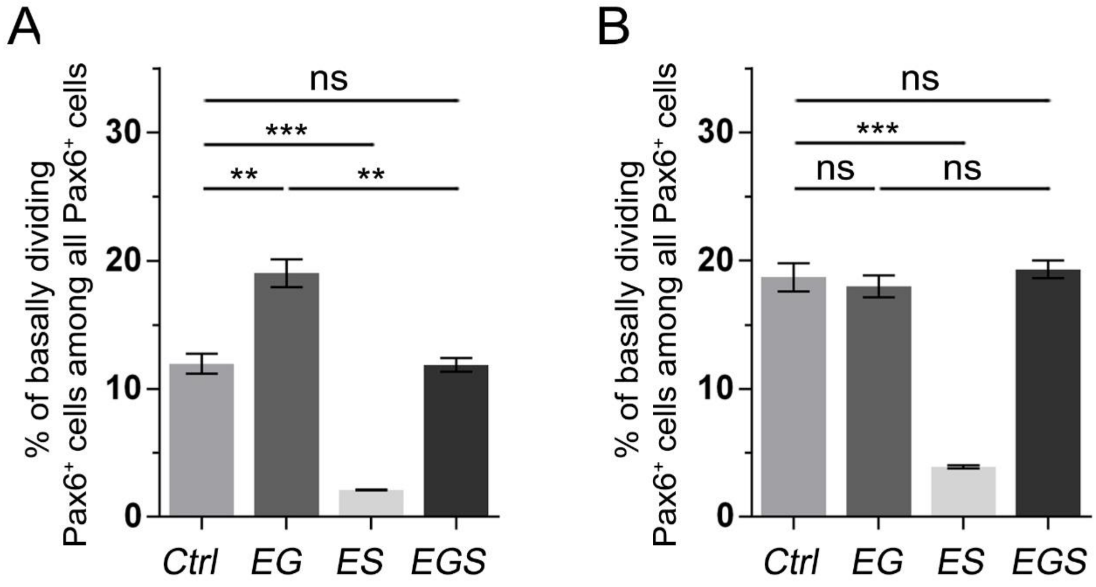
A
% of basally dividing
Pax6⁺ cells among all Pax6⁺ cells
ns
***
**
**
30
20
10
0
Ctrl
EG
ES
EGS
B
% of basally dividing
Pax6⁺ cells among all Pax6⁺ cells
ns
***
ns
ns
30
20
10
0
Ctrl
EG
ES
EGS

FIGURE S4

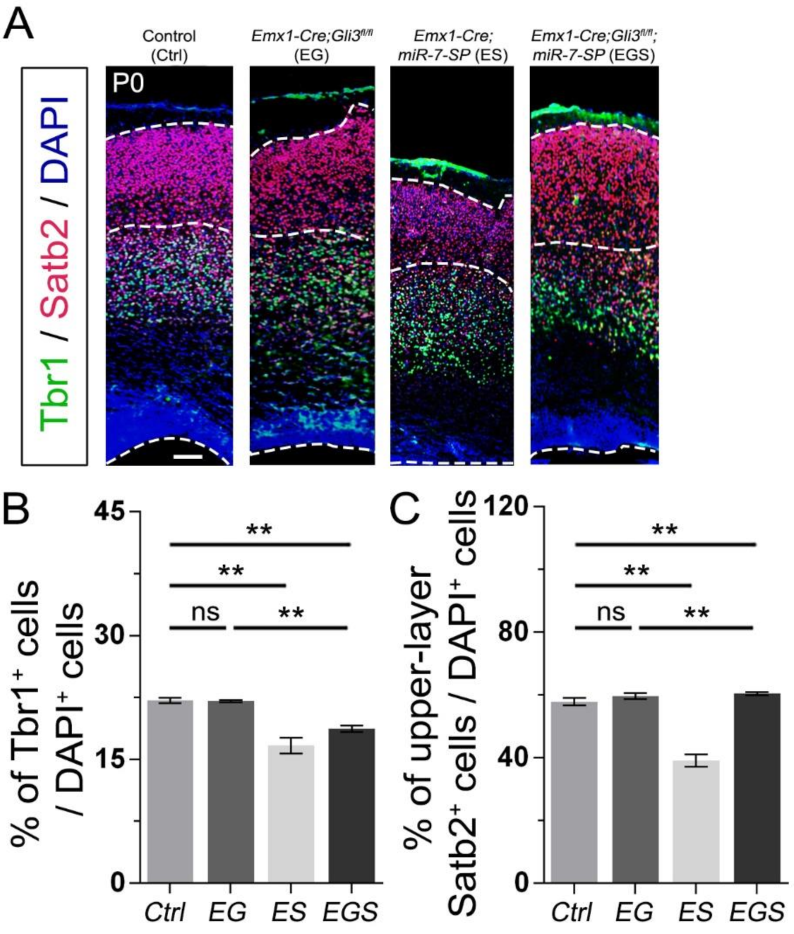
A
Control
(Ctrl)
Emx1-Cre;Gli3fl/fl
(EG)
Emx1-Cre;
miR-7-SP (ES)
Emx1-Cre;Gli3fl/fl;
miR-7-SP (EGS)
Tbr1 / Satb2 / DAPI
P0
B
% of Tbr1+ cells / DAPI+ cells
45
30
15
0
ns
**
Ctrl
EG
ES
EGS
C
% of upper-layer Satb2+ cells / DAPI+ cells
120
80
40
0

FIGURE S5

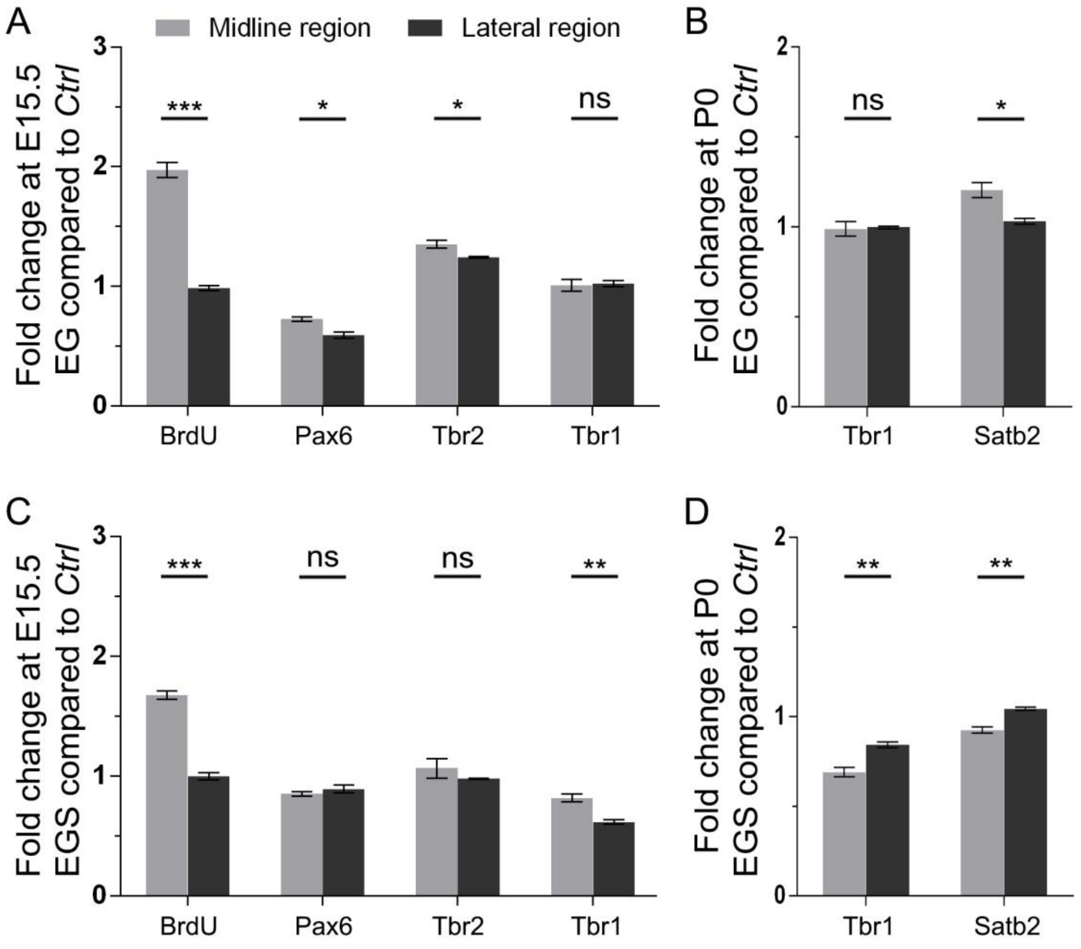

A
Midline region
Lateral region
Fold change at E15.5 EG compared to Ctrl
3
2
1
0
***
*
*
ns
BrdU
Pax6
Tbr2
Tbr1
B
Fold change at P0 EG compared to Ctrl
2
1
0
ns
*
Tbr1
Satb2
C
Fold change at E15.5 EGS compared to Ctrl
3
2
1
0
***
ns
ns
**
BrdU
Pax6
Tbr2
Tbr1
D
Fold change at P0 EGS compared to Ctrl
2
1
0
**
**
Tbr1
Satb2

FIGURE S6

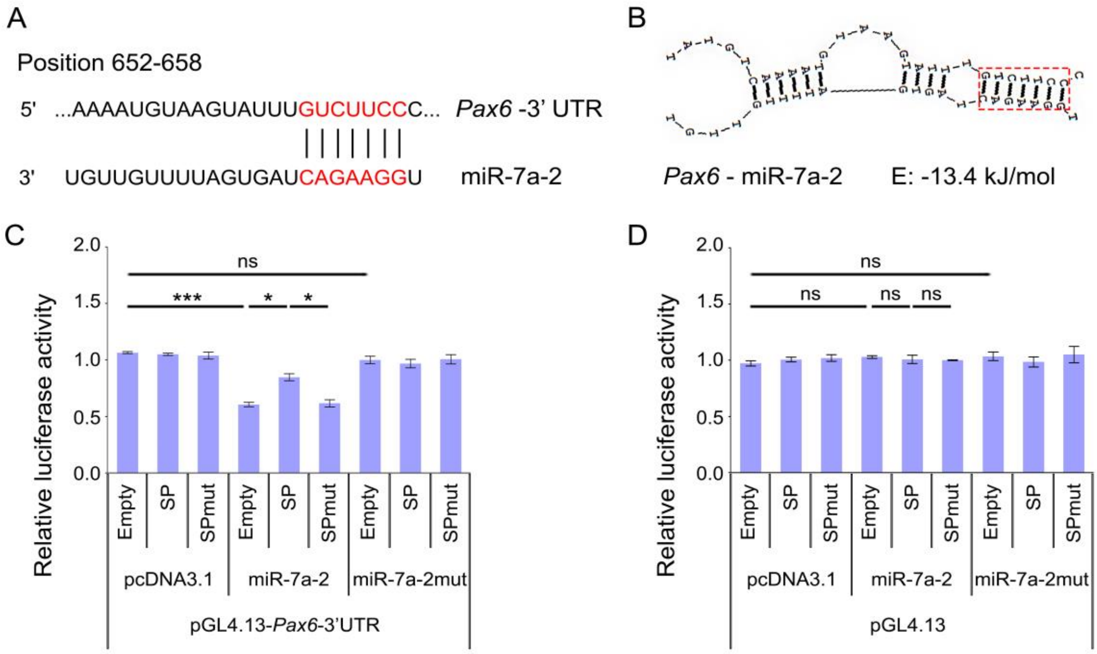
A
Position 652-658
5' ...AAAAUGUAAGUAUUUGUCUUCCC... Pax6 -3' UTR
3' UGUUGUUUUAGUGAUCAGAAGGU miR-7a-2
B
Pax6 - miR-7a-2 E: -13.4 kJ/mol
C
Relative luciferase activity
ns
***
*
*
Empty
SP
SPmut
pcDNA3.1
miR-7a-2
miR-7a-2mut
pGL4.13-Pax6-3'UTR
D
Relative luciferase activity
ns
pGL4.13
